# Supplementary material for: A CRISPRi screen in E. coli reveals sequence-specific toxicity of dCas9
Source: Nat Commun. 2018 May 15;9:1912. doi: 10.1038/s41467-018-04209-5 (PMC5954155; doi:10.1038/s41467-018-04209-5)
Supplement: Supplementary file 1 — Supplementary Information [file 41467_2018_4209_MOESM1_ESM.pdf]

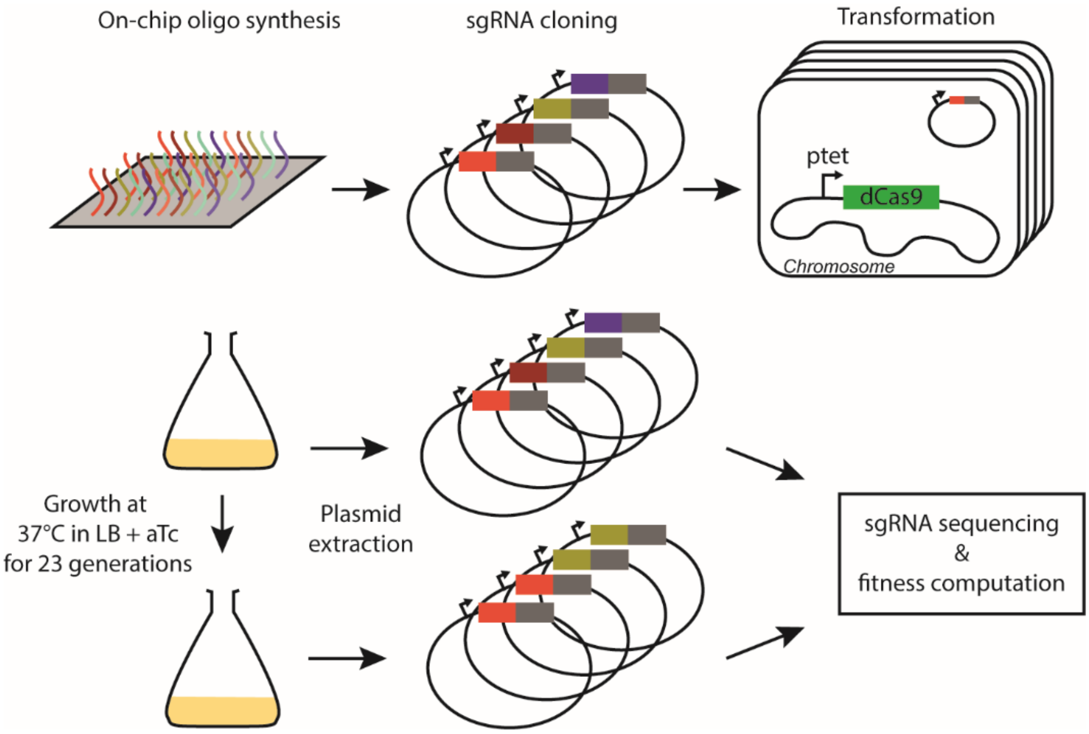

Supplementary Figure 1. Experimental scheme of the CRISPRi screen performed in *E. coli*. Oligonucleotides synthesized on chip were cloned on plasmid psgRNA and electroporated into *E. coli* LC-E18 carrying *dCas9* under the control of a Ptet promoter. Cells were grown at 37°C in LB supplemented with aTc and the psgRNA library was extracted and sequenced at the beginning and at the end of the experiment. Sequencing the guides enables to estimate their relative abundance in the population and to compute their fitness effect on the cell.

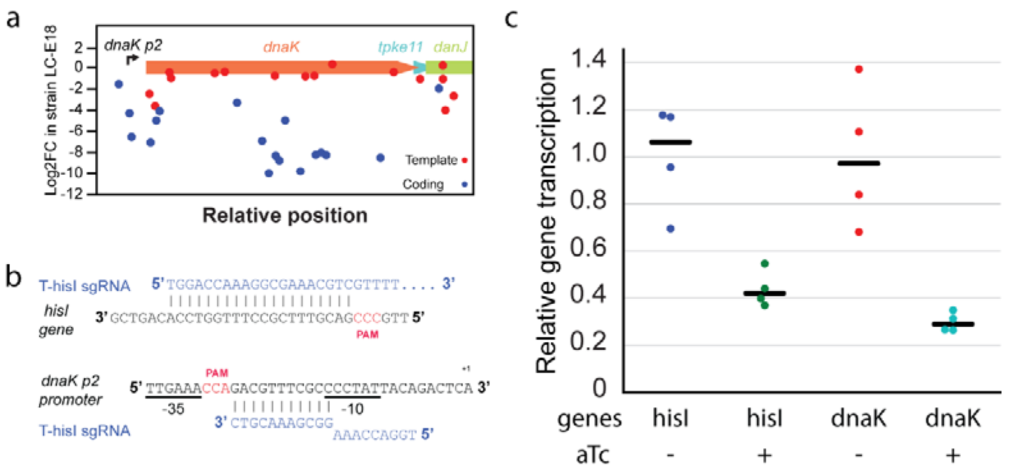

Supplementary Figure 2. The T-hisI guide RNA blocks the expression of the dnaK-dnaJ operon. (a) Fitness effect of guides targeting the dnaK-dnaJ operon (red: template strand, blue: coding strand). (b) There is a 11 bp perfect match between the seed sequence of the T-hisI guide RNA and the dnaK p2 pormoter region. (c) RT-qPCR results showing repression of both *dnaK* and *hisI* by the T-hisI guide RNA, after 2H of growth with and without aTc. n = 4, the black bar shows the median.

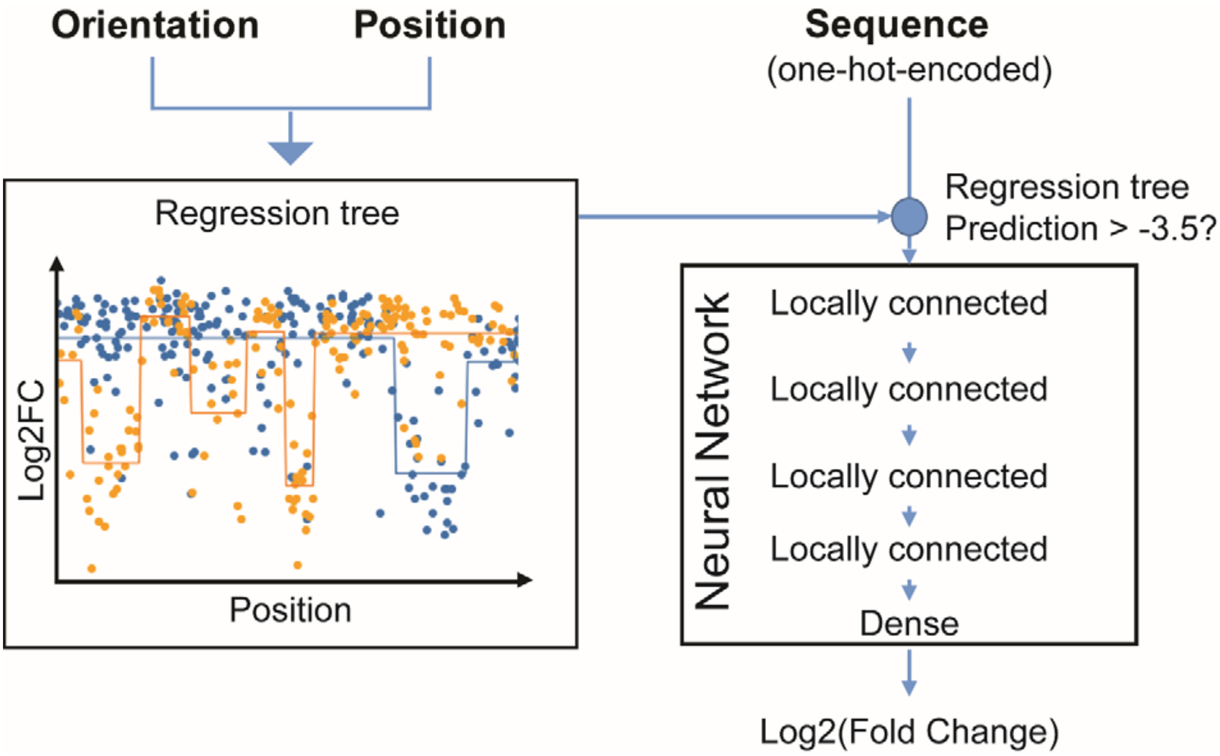

Supplementary Figure 3. Flow chart of machine learning analysis. A regression tree was first fitted using guide orientation and position as the unique features to predict log2FC. This enables to identify regions and orientations were guides consistently produce a fitness defect. The goal of this analysis was then to investigate the fitness defect produced by guides that target in “neutral” regions. In these regions, the log2FC of guides is not consistent and most guides have no effect. We kept guides in genomic regions where the prediction fo the regression tree was greater than -3.5 and then fitted a neural network using the one-hot-encoded sequence as the unique feature to predict the log2FC. The neural network consists in 4 locally connected layers with a kernel size of 5 followed by a dense layer. More details are provided in the methods section.

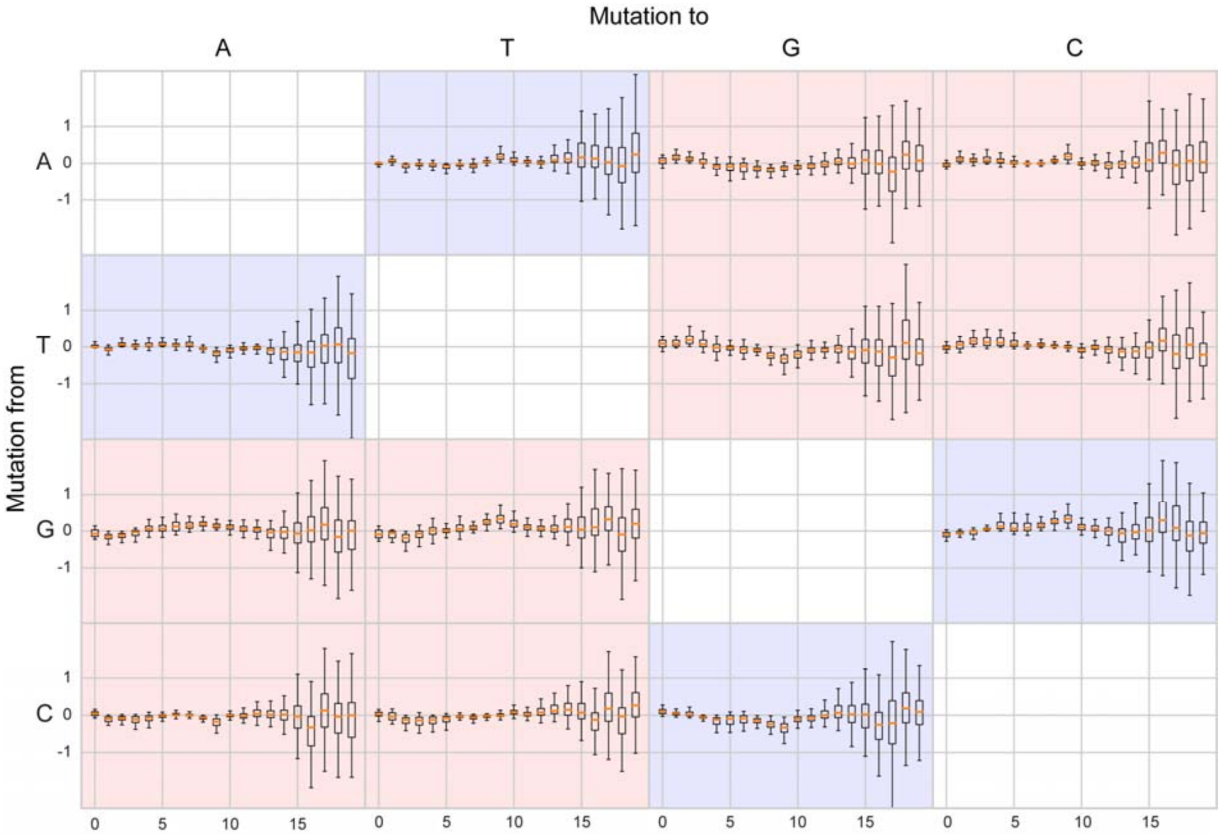

Supplementary Figure 4. Effect of mutations on the model predictions. We generated 1000 random sequences and computed the effect on the model prediction or mutating each base to all possible bases. The distribution of these effects are shown as boxplots for each type of mutation (using the default matplotlib boxplot parameters). The y-axis shows the difference in predicted log2FC between the mutated sequence and the initial sequence (a lower value means that the mutation is predicted to cause a stronger fitness defect). The x-axis shows the position along the guide sequence. Transitions are highlighted in blue and transversions in red.

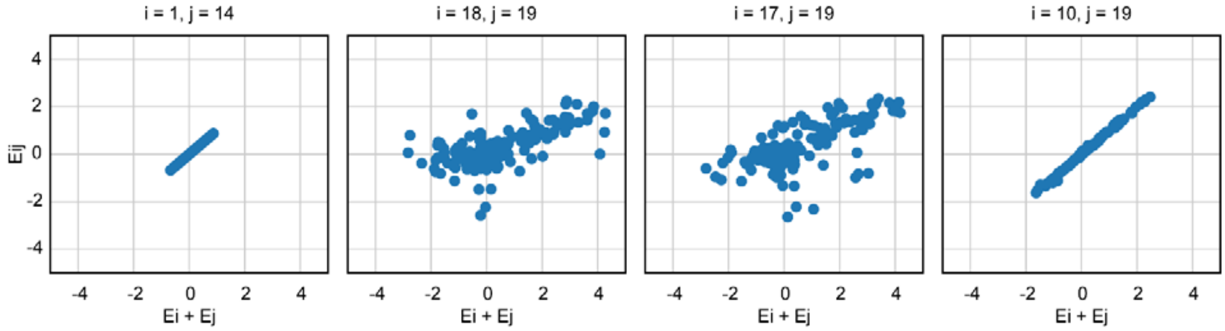

Supplementary Figure 5. Measuring interactions between positions in the model. To measure the level of interaction between positions we generated all possible pairs of mutations for each sequence in a set of 100 random sequences, and compared the effect of individual mutations to that of pairs of mutations. Positions are interacting if the effect of a double mutation (Eij) is different from the sum of the effect of the single mutations (Ei+Ej). As an example we plot Eij vs. Ei+Ej for interacting (i=18,j=19 ; i=17,j=19) and non-interacting positions (i=1,j=14; i=10,j=19).

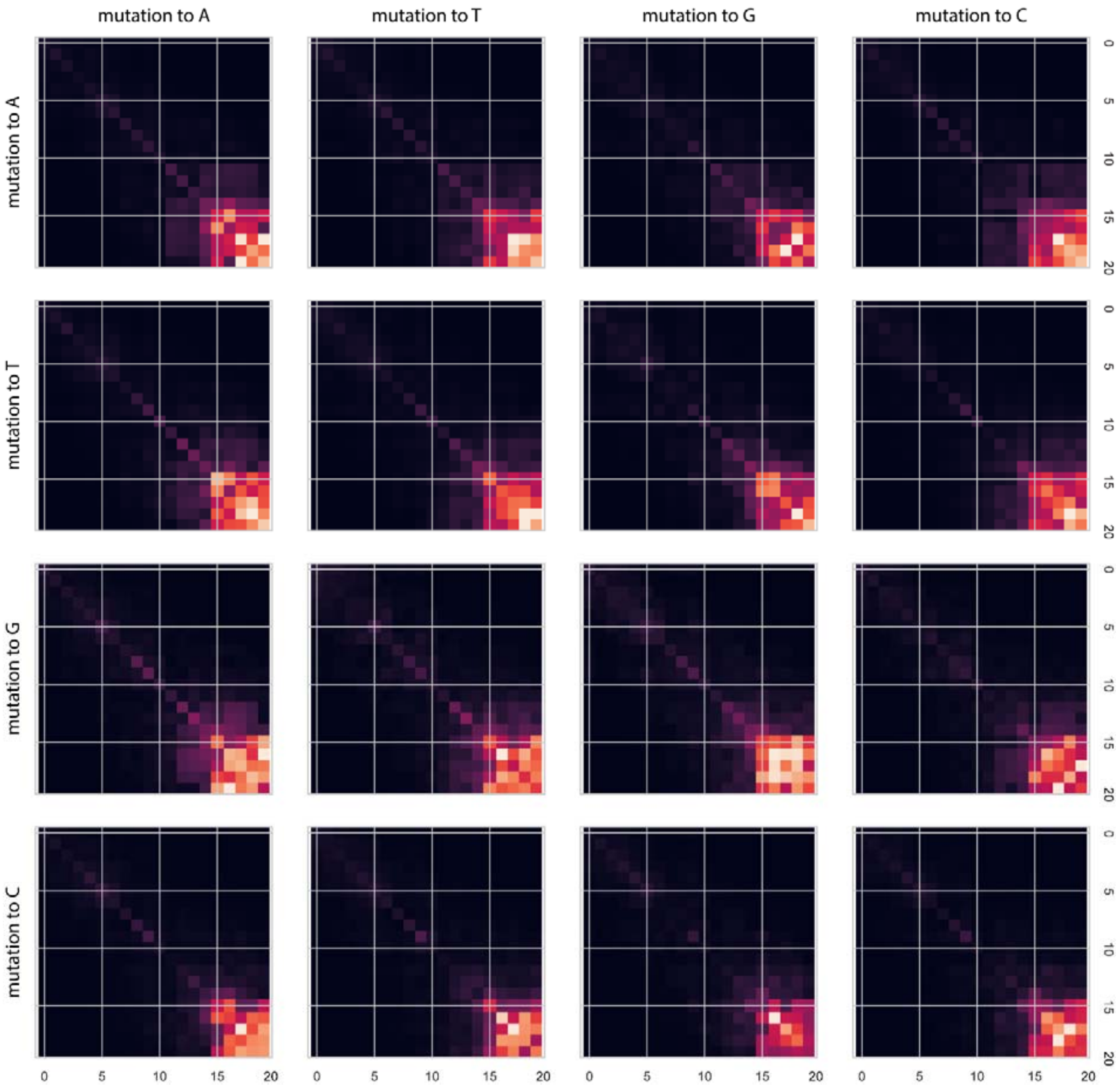

Supplementary Figure 6. Level of interaction predicted by the model for different combinations of mutations. We generated all possible pairs of mutations for each sequence in a set of 100 random sequences, and compared the effect of individual mutations to that of pairs of mutations. The color shows the average Euclidian distance between the effect of a double mutation and the sum of the effect of single mutations (dark: no interaction, white: strong interaction).

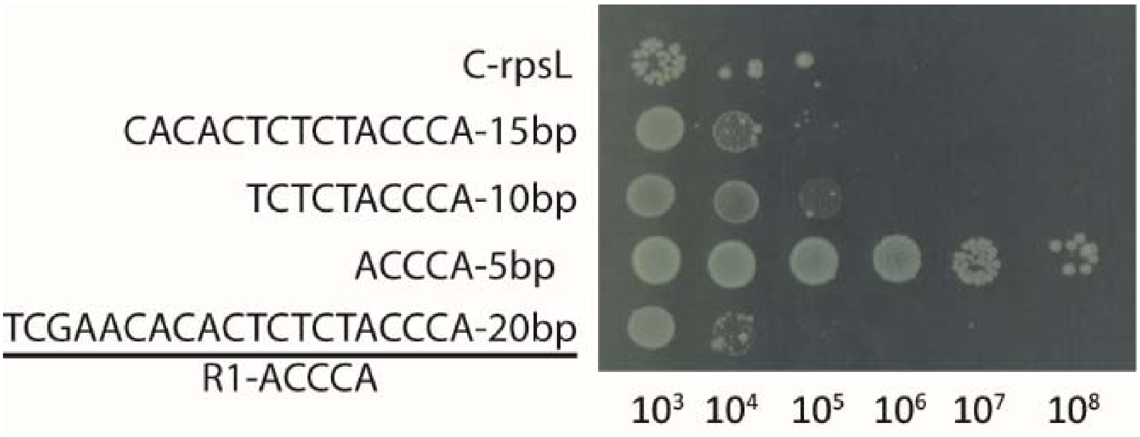

Supplementary Figure 7. Truncated guides with a bad seed sequence can still kill *E. coli*. Truncated single guide RNAs with the ACCCA seed sequence were cloned on plasmid psgRNA and transformed in strain LC-E18. Serial dilutions were then plated on LB supplemented with aTc and kanamycin. The C-rpsL lane shows a positive control where the transcription of *rpsL* essential gene is repressed by dCas9. n = 3.

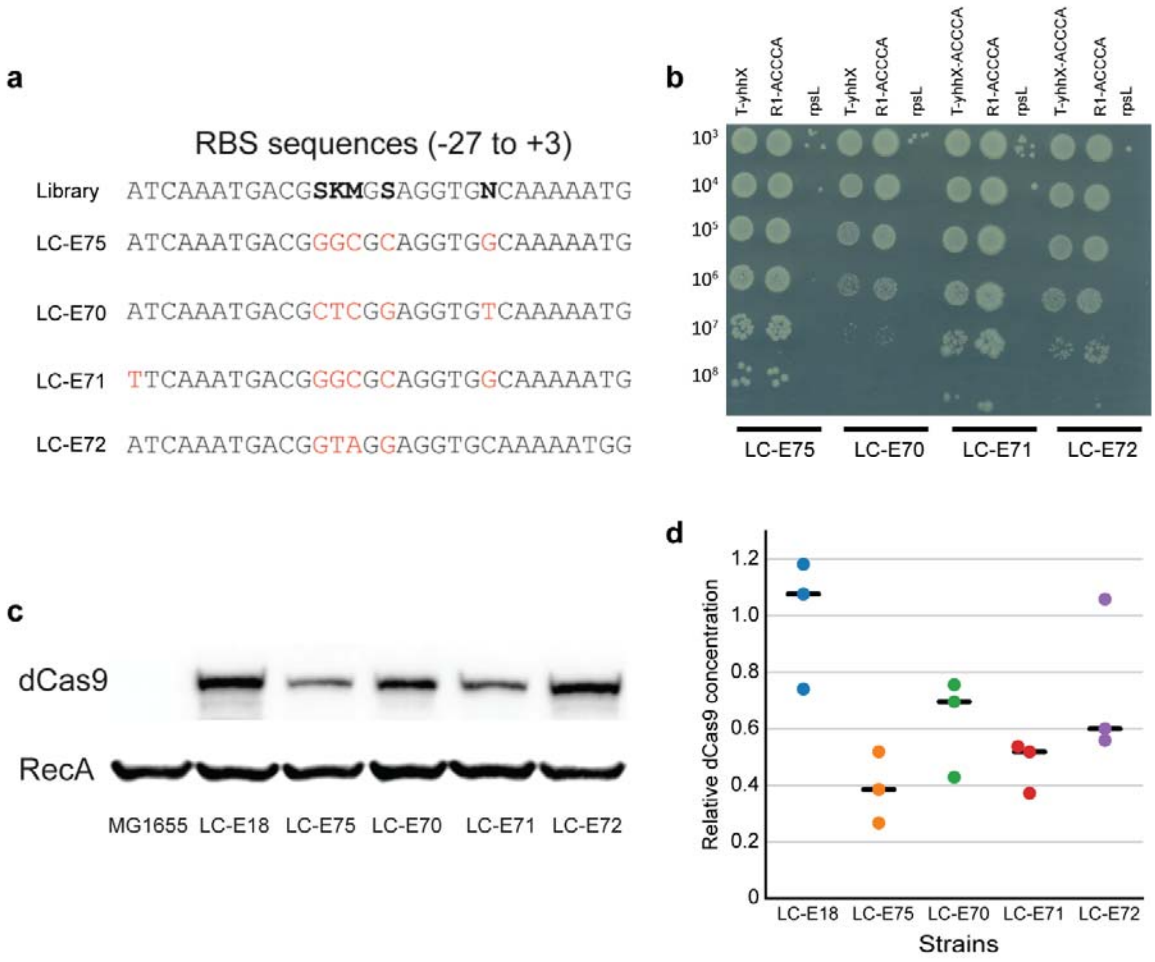

Supplementary Figure 8. The bad seed effect can be alleviated by reducing dCas9 concentration. A library of RBS controlling the expression of dCas9 was generate and clones were selected for their ability to survive the dCas9 expression in the presence of a bad seed sequence while still efficiently blocking the expression of *rpsL* when guided by the C-rpsL sgRNA. Four clones where selected (LC-E70, LC-E71, LC-E72 and LC-E75). (a) RBS sequence of the selected clones. (b) Cells carrying the T-yhhX, R1-ACCCA or C-rpsL guide RNAs were grown overnight, followed by serial dilution and plating with aTc. (c) Western blot image of selected dCas9 strains. (d) quantification results of 3 western blots, the black bar shows the median (Uncorpped photos are shown in Supplementary Figure 16).

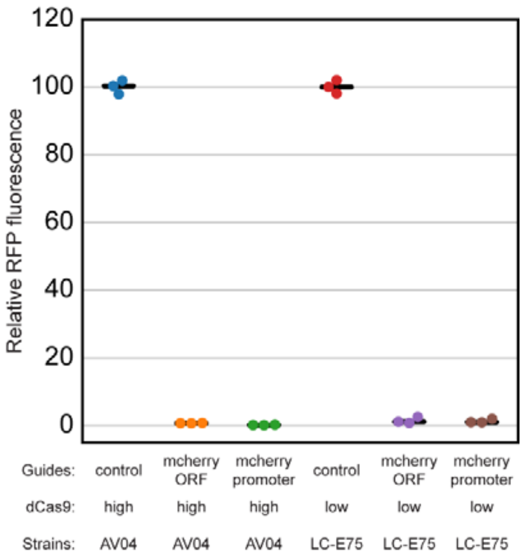

Supplementary Figure 9. The LC-E75 strain efficiently blocks target gene expression. Strain LC-E75 carries an optimized dCas9 expression cassette integrated at the 186 attB site and a constitutively expressed *mcherry* reporter gene at the lambda attB site. Strain AV04 is identical to LC-E75 but carries the stronger un-optimized dCas9 expression cassette from strain LC-E18. Cells carrying plasmids (psgRNA::C-mcherry20orf, psgRNA::C-mcherry20p) or a control psgRNA were grown with aTc overnight. The next morning, the cells were diluted 250 times in fresh medium with 1 nM aTc and kanamycine, and grown for 1.75 hours at 37°C to reach the exponential phase. The cells were fixed and fluorescence was measured using flow cytometry. n = 3.

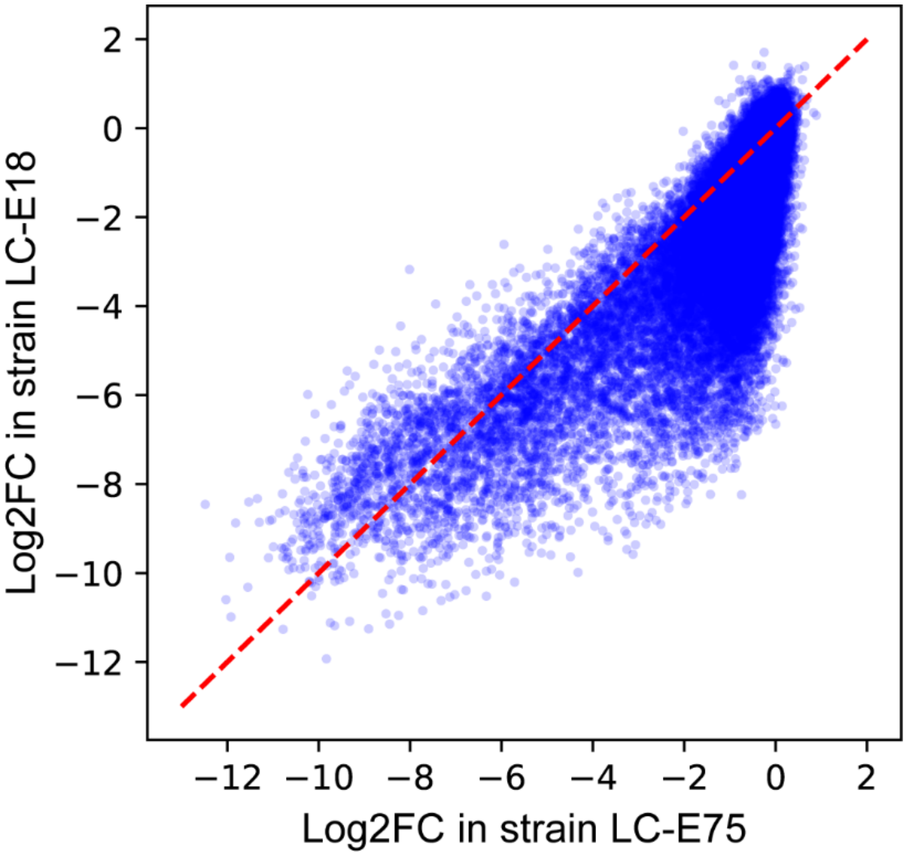

Supplementary Figure 10. Comparision of fitness measurments in strain LC-E18 and LC-E75. Scatter plot of the log2FC value (LC-E18 vs LC-E75) for all the guides in the library.

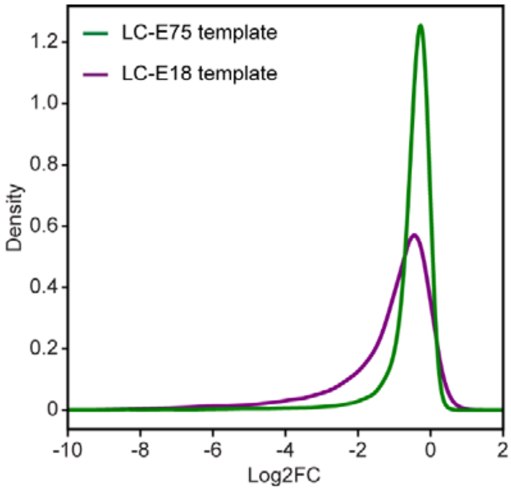

Supplementary Figure 11. Comparison of the fitness effects of sgRNAs targeting the template strand of genes. The log2FC value distribution of guides targeting template strand is much narrower in strain LC-E75, showing that guide RNAs have a more consistent effect in strain LC-E75 than LC-E18.

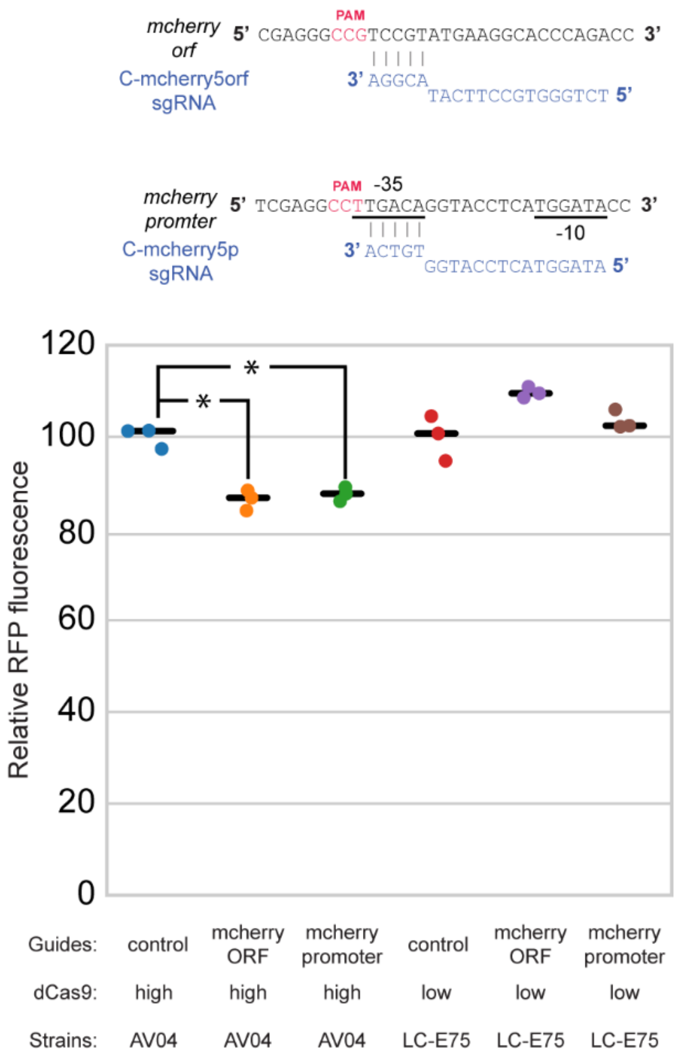

Supplementary Figure 12. A 5 bp of seed match has minimal or no effect on gene transcription. Guides with only 5 bp of identity between the PAM-proximal region and the target where used to target either the mcherry ORF region (C-mcherry5orf) or the promoter region (C-mcherry5p). Strain LC-E75 carries an optimized dCas9 expression cassette integrated at the 186 attB site and a constitutively expressed *mcherry* reporter gene at the lambda attB site. Strain AV04 is identical to LC-E75 but carries the stronger un-optimized dCas9 expression cassette from strain LC-E18. Cells were grown with aTc overnight. The next morning, the cells were diluted 250 times in fresh medium with 1 nM aTc and kanamycine, and grown for 1.75 hours at 37°C to reach the exponential phase. The cells were fixed and fluorescence was measured using flow cytometry. The In the high dCas9 concentraction strain (AV04), 5 bp matches can block transcription by 13.7% and 12.5% when targeting the ORF and promoter region respectively. In the low dCas9 concentraction strain (LC-E75), repression was not significant. "*" indicates a p-test value than 0.05 (double sided t-test with equal variance). n = 3, the black bars show the median.

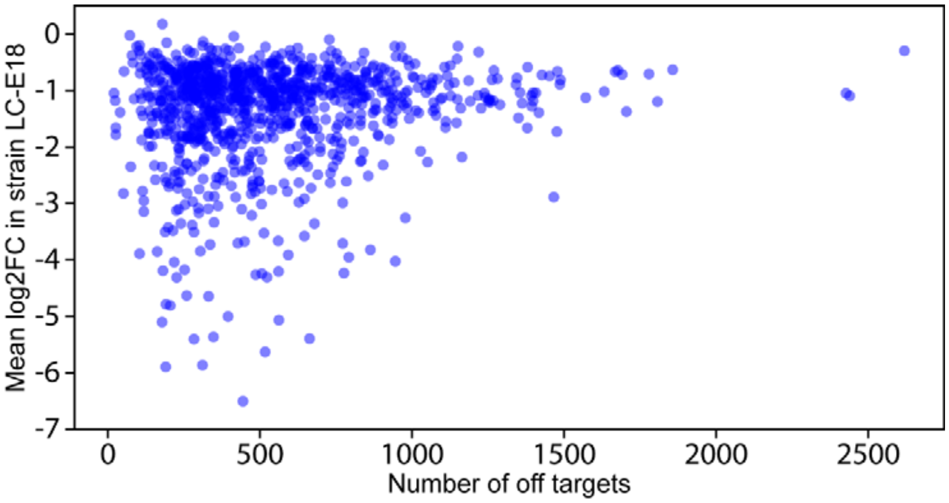

Supplementary Figure 13. The bad seed effect does not correlate with the number of off-targets in the genome. Mean log2FC for guides sharing the same 5nt seed sequence as a function of the number of off-targets in the genome of *E. coli* MG1655 that have a perfect match to these 5nt and a “NGG” PAM.

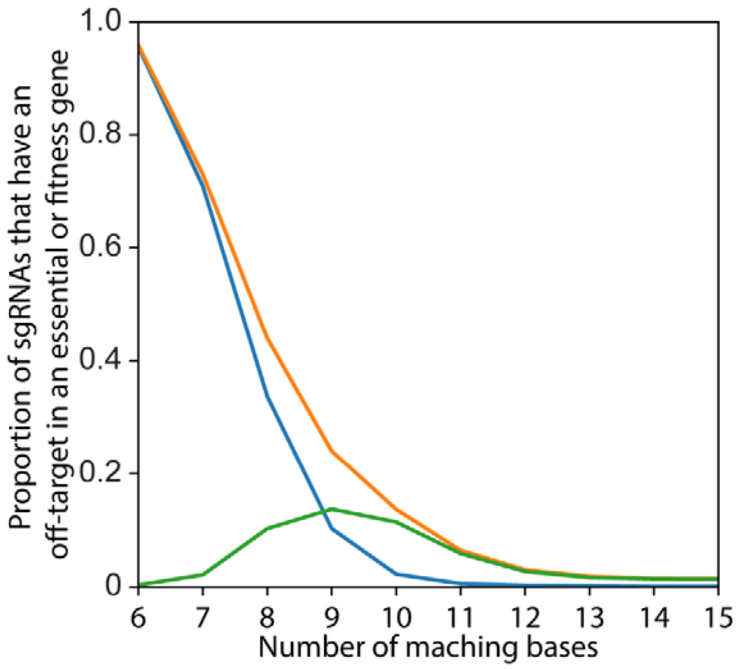

Supplementary Figure 14. Guide RNAs with more than 9nt of perfect identity between the seed sequence and off-target positions can have a strong fitness effect. We plot here the proportion of guide RNAs which have an off-target position in a region where guides consistently produce a strong fitness defect. This proportion is shown for guides that have an unexpectedly strong log2FC (<-3.5) while targeting the template strand of non-essential genes (orange), as well as for guides targeting the same regions but with log2FC>-3.5 (blue), which can be interpreted as the false discovery rate. The green curve is the difference between the green and blue curves. The maximum is at 9nt, which indicates that 9nt of identity in the seed sequence is enough to produce a strong fitness defect, but mostly false positive off-target positions are detected when going down to 8nt of identity.

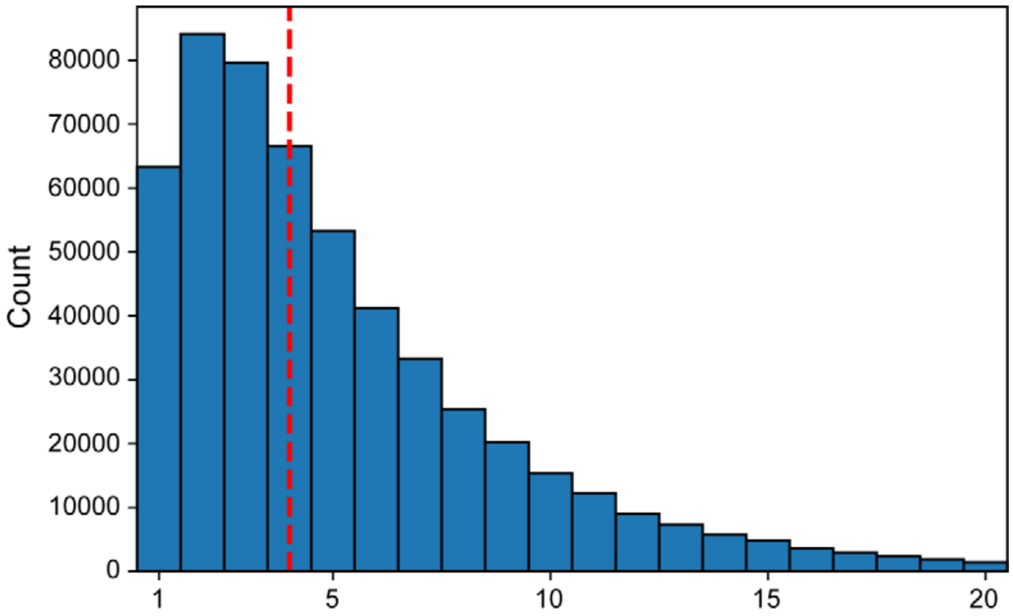

Supplementary Figure 15. Distribution of the number of off-target for guides targeting the chromosome of *E. coli* MG1655. 88.3% of all possible guides with a target in the chromosome of *E. coli* MG1655 have at least one off-target with 9nt of identity in the seed sequence or more. The red dash line is the medium value (4) of the distribution.

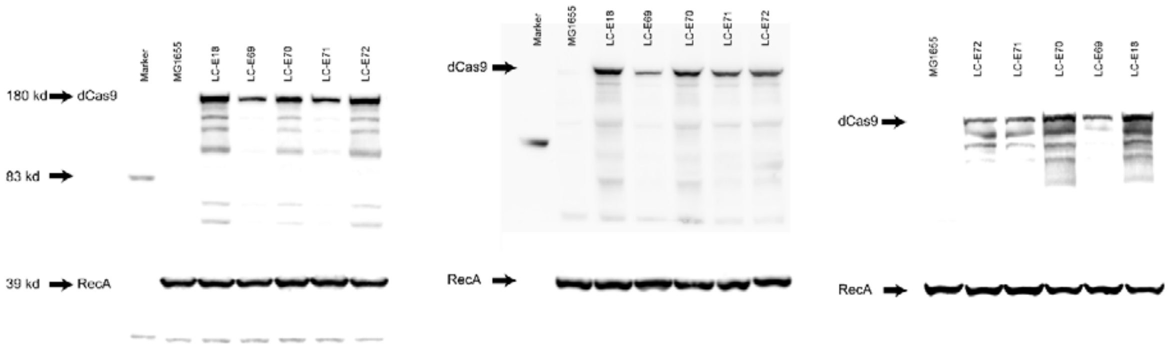

Supplementary Figure 16. Uncropped western blot photos. Uncropped photos of 3 westernblot repeats.

Supplementary Table 1. Repartition of guides according to their fitness effect in strain LCE-18 and binding orientation.

	Template strand		Coding strand		All
**Log2FC**	**Log2FC>-3.5**	**Log2FC<-3.5**	**Log2FC>-3.5**	**Log2FC<-3.5**	
**Essential**	2628	351	701	1834	5514
**Non-essential**	33612	2499	25989	2694	64794
**All**	36240	2850	26690	4528	70308

Supplementary Table 2. Mutations selected in strain LC-E18 that abolish the fitness defect produced by the T-yhhX-ACCCA guide RNA while maintaining strong repression of *rpsL*. Mutations in samples 1,2,3,4 and 6 led to frameshifts in the *dCas9* gene. The ability of these strains to still efficiently block *rpsL* shows that they still express low levels of dCas9 likely thanks to translational slippage.

Sample ID	Mutation types	Mutations	Consequences
**1**	Insertion	T inserted between 423 and 424	Frame shift
**2**	Insertion	T inserted between 1714 and 1715	Frame shift
**3**	Insertion	T inserted between 1714 and 1715	Frame shift
**4**	Insertion	T inserted between 1714 and 1715	Frame shift
**5**	Deletion	Deletion from -27 to +7	Promoter mutated
**6**	Insertion	T inserted between 1714 and 1715	Frame shift

Supplementary Table 3. Plasmids constructed in this study.

Plasmid name	Backbone			Fragment 1			Fragment 2		
	Template	Primer 1	Primer 2	Template	Primer 1	Primer 2	Template	Primer 1	Primer 2
pCRRNAcos	pCRRNA digested with AvrII	N/A	N/A	MG1655, lambda lysogen	LC74	LC75	N/A	N/A	N/A
psgRNA*	pCRRNAcos	LC41	LC42	sgRNA_bsaI_gBlock (IDT)	N/A	N/A	N/A	N/A	N/A
psgRNAc*	pCas9	LC89	LC132	psgRNAcos	LC316	LC317	N/A	N/A	N/A
psgRNAcos::R1_ACCCA_5bp	psgRNAcos	LC191	LC1027	psgRNAcos	LC192	LC1028	N/A	N/A	N/A
psgRNAcos::R1_ACCCA_10bp	psgRNAcos	LC191	LC1029	psgRNAcos	LC192	LC1030	N/A	N/A	N/A
pOSIP-KL-sulA-GFP	pOSIP-KL digested with EcoRI and PstI	N/A	N/A	pZA31-sulA-GFP	LC245	LC246	N/A	N/A	N/A
pOSIP-KL-mcherry	pOSIP-KL digested with EcoRI and PstI	N/A	N/A	pDB127	V1	V40	pFB262	V38	V39
pOSIP-KH-RBS2-dCas9	pOSIP-KH digested with EcoRI and PstI	N/A	N/A	pDB275	LC100	LC283	pdCas9-bacteria	LC284	LC285
pOSIP-KO-RBS2-dCas9	pOSIP-KO digested with EcoRI and PstI	N/A	N/A	pOSIP-KH-RBS2-dCas9	LC124	LC125	N/A	N/A	N/A
pOSIP-CO-RBS-library-dCas9	pOSIP-CO digested with BamHI and PstI	N/A	N/A	pDB275	LC653	LC1090	pdCas9-bacteria	LC1088	LC285

* Sequence provided in Supplementary Data 4

Plasmids and strains were deposited in Addgene.

Supplementary Table 4. Primers used for cloning.

Name	Sequence (5' to 3')
LC41	TGCAGCGCGATCGTAATCAGGATCCCATGGTACGCGT
LC42	ACAGAACTTAATGGGCCCGAAGACGAAAGGGCCTCGT
LC74	TGAACGCTCTCCTGAGTAGGACAAATCCGCCGCCCTAGACCCTCCACGCACGTTGTGATATG
LC75	CCTTTGAGTGAGCTGATACCGCTCGCCGCAGCCGAACGCCCCAAAAAGCCTCGCTTTCAGC
LC89	CAGGTGCTACATTTGAAGAGAT
LC100	GCAGGACGCCCGCCATAAACTGCCAGGAATTGGGGATCGGTTAAGACCCACTTTCACATTTAAG
LC124	CTGCCAGGAATTGGGGATC
LC125	CAGTTTAGGTTAGGCGCCAT
LC132	GGGGAGAGCCTGAGCAAA
LC191	GTCTAGGGCGGCGGATTTG
LC192	CGCTCTCCTGAGTAGGACAAAT
LC245	GACGCCCGCCATAAACTGCCAGGAATTGGGGATCGGATCGGTATTCAATTGTGCCCA
LC246	TAGGTTAGGCGCCATGCATCTCGAGGCATGCCTGCATTATTTGTATAGTTCATCCATGCCA
LC283	TCCATTTTTGCCTCCTAACTAGGTCATTTGATATGCCTCC
LC284	CCTAGTTAGGAGGCAAAAATGGATAAGAAATACTCAATAGGC
LC285	AGTTTAGGTTAGGCGCCATGCATCTCGAGGCATGCCTGCAATGCCTGGAGATCCTTACTC
LC293	GTTTTAGAGCTAGAAATAGCAAGTTAA
LC294	ACTAGTATTATACCTAGGACTGAGCTA
LC296	TATATTTTAGGAATTCTAAAGATCTTTGACAGCTAGCTCAGTCCTAGGTATAATACTAGT
LC297	ACTTTTTCAAGTTGATAACGGACTAGCCTTATTTTAACTTGCTATTTCTAGCTCTAAAAC
LC316	CAAATGCCTGAGGCCAGTTTGCTCAGGCTCTCCCCCCTGATTACGATCGCGCTG
LC317	TTCAGTGCAATTTATCTCTTCAAATGTAGCACCTGGCTAGGAGGTGACTGAAGT
LC499	GATCGGAAGAGCACACGTCTGAACTCCAGTCAC
LC609	GCACGCCCGTCGCTCAGTCCTAGGTATAATACTA
LC610	TATTATACCTAGGACTGAGCGACGGGCGTGC
LC653	GGAATTGGGGATCGGAATTCGAGCTCGGTACCCGGGGGTTAAGACCCACTTTCACATT
LC961	GCACAAAGTCAGACTTTATCTATATTTGTAAGGGGTGAATCTTGATGACAACATGGGAATTAGCCATGGTCC
LC962	TGTAGGGTCGGAGCGTTAACGTTGCCAGAGGCGCTGGAGTACAGCACGTAGTGTAGGCTGGAGCTGCTTC
LC1027	TAATACTAGTTCTCTACCCAGTTTTAGAGCTAGAAATAGCAAG
LC1028	CTAGCTCTAAAACTGGGTAGAGAACTAGTATTATACCTAGGACTGAGCTA
LC1029	TAATACTAGTACCCAGTTTTAGAGCTAGAAATAGCAAG
LC1030	CTAGCTCTAAAACTGGGTACTAGTATTATACCTAGGACTGAGCTA
LC1088	TGATAGAGTGATATCCGGAGGCATATCAAATGACGSKMGSAGGTGNCAAAAATGGATAAGAAATACTCAATAGG CTTAGCT
LC1090	CATTTGATATGCCTCCGGATATCACTCTATCA
V1	CGCCATAAACTGCCAGGAATTGGGGATCGGCCTATGAACTGTCGACTCGAGG
V38	GGAGAAATCTAGATGGTTTCCAAGGGCGAGGAGGAT
V39	TTAGGCGCCATGCATCTCGAGGCATGCCTGCATTATTTGTACAGCTCATCCATGCC
V40	ATCCTCCTCGCCCTTGGAAACCATCTAGATTTCTCCTCTTTAAAGGAATTCC

Supplementary Table 5. Plasmids used in this study.

Plasmids from other studies	Addgene Catalog # (or References)
pCRRNAcos	Plasmid #78493
pCas9	Plasmid #42876
pOSIP-KH	Plasmid #45983
pOSIP-KL	Plasmid #45984
pOSIP-KO	Plasmid #45985
pOSIP-CO	Ref[1]
pZA31-sulA-GFP	Plasmid #78492
pDB127	Ref[2]
pDB275	Ref[3]
pdCas9-bacteria	Plasmid #44249
pE-FLP	Plasmid #45978
pKD3	Plasmid #45604
pKOBEG-A	Ref[4]
pFB262	Ref[5]

Supplementary Table 6. sgRNA guide sequences and corresponding primers

Target name	Target sequences	Forward primer	Reverse primer
**T-lpoB**	TTGTCGCCGCAGGACAGT TT	TAGTTTGTCGCCGCAGGACAGT TT	AAACAAACTGTCCTGCGGCGACA A
**T-lpoB2**	ATTCGCCATTATGACTGG AA	TAGTATTCGCCATTATGACTGG AA	AAACTTCCAGTCATAATGGCGAA T
**C-lopB**	CGCCATCGACAGTTGCTG GG	TAGTCGCCATCGACAGTTGCTG GG	AAACCCCAGCAACTGTCGATGGC G
**T-hisI**	TGGACCAAAGGCGAAAC GTC	TAGTTGGACCAAAGGCGAAAC GTC	AAACGACGTTTCGCCTTTGGTCC A
**C-hisI**	GGTGACTTTGCCGCTTTC GA	TAGTGGTGACTTTGCCGCTTTC GA	AAACTCGAAAGCGGCAAAGTCAC C
**C-rpsL**	AGCGCGGAGTTCGGTTTT TT	TAGTAGCGCGGAGTTCGGTTTT TT	AAACAAAAAACCGAACTCCGCGC T
**R1-ACCCA**	TCGAACACACTCTCTACC CA	TAGTTCGAACACACTCTCTACC CA	AAACTGGGTAGAGAGTGTGTTCG A
**R1-ACCCA**	GCTACCTTAACGCCTACC CA	GCTACCTTAACGCCTACCCA	AAACTGGGTAGGCGTTAAGGTAG C
**T-yhhX**	TTGTATCAAACCATCACC CA	TAGTTTGTATCAAACCATCACC CA	AAACTGGGTGATGGTTTGATACA A
**T-ydeO**	ACATGAAGCCGGCGCAC CCA	TAGTACATGAAGCCGGCGCAC CCA	AAACTGGGTGCGCCGGCTTCATG T
**R1-TGGAA**	CCGCTATGTCAGGCGTG GAA	TAGTCCGCTATGTCAGGCGTG GAA	AAACTTCCACGCCTGACATAGCG G
**R2-TGGAA**	GTCAGTCATATTAACTGG AA	TAGTGTCAGTCATATTAACTGG AA	AAACTTCCAGTTAATATGACTGAC
**T-garD**	TGAGCGCCTGCTGACTG GAA	TAGTTGAGCGCCTGCTGACTG GAA	AAACTTCCAGTCAGCAGGCGCTC A
**R1-ACCCA_15bp**	CACACTCTCTACCCA	TAGTCACACTCTCTACCCA	AAACTGGGTAGAGAGTGTG
**T-bioC**	ACGCGGGTTGTGGACCT GGC	TAGTACGCGGGTTGTGGACCT GGC	AAACGCCAGGTCCACAACCCGC GT
**C-mcherry20orf**	TCTGGGTGCCTTCATACG GA	TAGTTCTGGGTGCCTTCATACG GA	AAACTCCGTATGAAGGCACCCAG A
**C-mcherry20p**	TATCCATGAGGTACCTGT CA	TAGTTATCCATGAGGTACCTGT CA	AAACTGACAGGTACCTCATGGAT A
**C-mcherry5orf**	AGACCCACGGAAGTAACG GA	TAGTAGACCCACGGAAGTAACG GA	AAACTCCGTTACTTCCGTGGGTC T
**c-mcherry5p**	ATAGGTACTCCATGGTGT CA	TAGTATAGGTACTCCATGGTGT CA	AAACTGACACCATGGAGTACCTA T

Supplementary Table 7. Strains made using the OSIP system.

Strain Name	Original strain	Integration at lambda attB	Integration at primary 186 attB	Integration at HK022 attB
**LC-E18**	MG1655	pOSIP-KL-sulA-GFP	N/A	pOSIP-KH-RBS2-dCas9
**LC-E75**	MG1655	pOSIP-KL-mcherry	pOSIP-CO-RBS-library-dCas9 (2-3)*	N/A
**AV04**	MG1655	pOSIP-KL-mcherry	pOSIP-KO-RBS2-dCas9	N/A

*The number in the parentheses is corresponding to the selected colony number.

Supplementary Table 8. Primers and corresponding indexes used to prepare the sequencing libraries.

Primer name	Index	primer sequences	PCR function
LC606	AGAT	TTCCCTACACGACGCTCTTCCGATCTTAGANNNNGCACGCCCGTCGCTCA GTCCTAGGTATAATACTA	1st PCR forward
LC607	TCTC	TTCCCTACACGACGCTCTTCCGATCTCTCTNNNNGCACGCCCGTCGCTCA GTCCTAGGTATAATACTA	1st PCR forward
LC608	CTTA	TTCCCTACACGACGCTCTTCCGATCTATTCNNNNGCACGCCCGTCGCTCA GTCCTAGGTATAATACTA	1st PCR forward
LC863	N/A	GTGACTGGAGTTCAGACGTGTGCTCTTCCGATCTNNNNNNAAAGGACCC GTAAAGTGATAATGAT	1st PCR reverse
LC415	N/A	AATGATACGGCGACCACCGAGATCTACACTCTTTCCCTACACGACGCT	2nd PCR forward
LC416	CGAGTAAT	CAAGCAGAAGACGGCATACGAGATCGAGTAATGTGACTGGAGTTCAGAC G	2nd PCR reverse
LC417	TCTCCGGA	CAAGCAGAAGACGGCATACGAGATTCTCCGGAGTGACTGGAGTTCAGAC G	2nd PCR reverse
LC420	TTCTGAAT	CAAGCAGAAGACGGCATACGAGATTTCTGAATGTGACTGGAGTTCAGACG	2nd PCR reverse
LC421	ACGAATTC	CAAGCAGAAGACGGCATACGAGATACGAATTCGTGACTGGAGTTCAGAC G	2nd PCR reverse

Supplementary Table 9. qPCR primers and probes.

**Targeted gene**	**rrsA**	**hisI**	**lpoB**	**bioC**	**lgt**
**Forward primer (5'----- 3')**	GGATAACTACTGG AAACGGTAGC	CAGTGGCTGTTCC TGTATCAA	CGCTCATTACGTG CTGTACT	GATATCGAATCCC TGCCGTTAG	GCCACCCATCACA GCTTTA
**Reverse primer (5'----- 3')**	CTAATCCCATCTG GGCACATC	ATACAGTTTGGCG GTGTAGG	CCAGATAATTTCG CCCGTCT	CGGAGTGCCGTG GATAAAT	GCCGTAACCAATC AGGAACA
**Probe (5'----- 3')**	FAM -- TACCGCATAACGT CGCAAGACCAA -- BHQ1	FAM -- CGCAAATCTGCCG ATCCGGAAAC-- BHQ1	FAM -- TAACGCTCCGACC CTACAAATGCA-- BHQ1	N/A	N/A

Supplementary References:

1. St-Pierre, F. *et al.* One-Step Cloning and Chromosomal Integration of DNA. *ACS Synth. Biol.* **2,** 537–541 (2013).
2. Jiang, W., Bikard, D., Cox, D., Zhang, F. & Marraffini, L. A. RNA-guided editing of bacterial genomes using CRISPR-Cas systems. *Nat. Biotechnol.* **31,** 233–239 (2013).
3. Depardieu, F. *et al.* A Eukaryotic-like Serine/Threonine Kinase Protects Staphylococci against Phages. *Cell Host Microbe* **20,** 471–481 (2016).
4. Chaveroche, M.-K., Ghigo, J.-M. & d'Enfert, C. A rapid method for efficient gene replacement in the filamentous fungus Aspergillus nidulans. *Nucleic Acids Res.* **28,** e97 (2000).
5. Teeffelen, S. van *et al.* The bacterial actin MreB rotates, and rotation depends on cell-wall assembly. *Proc. Natl. Acad. Sci.* **108,** 15822–15827 (2011).
