# Supplementary material for: A CRISPRi screen in E. coli reveals sequence-specific toxicity of dCas9
Source: Nat Commun. 2018 May 15;9:1912. doi: 10.1038/s41467-018-04209-5 (PMC5954155; doi:10.1038/s41467-018-04209-5)
Supplement: Supplementary file 3 — Description of Additional Supplementary Information [file 41467_2018_4209_MOESM3_ESM.pdf]

Description of Additional Supplementary Files

File Name: Supplementary Data 1
Description: List of 64 operons containing essential genes used to study the effect of dCas9 binding in promoter regions.

File Name: Supplementary Data 2
Description: List of genes used in the reverse polar effect analysis.

File Name: Supplementary Data 3
Description: List of seed sequences and their average fitness effect in strain LCE18 and LC-E75.

File Name: Supplementary Data 4
Description: Plasmid sequences.

File Name: Supplementary Data 5
Description: Screen results.

File Name: Supplementary Data 6
Description: Indices of the training, validation and test sets.
